# Acute Myopericarditis After the Third Vaccination of BNT162b2 in a Middle-Aged Man

**DOI:** 10.7759/cureus.28857

**Published:** 2022-09-06

**Authors:** Satoshi Nakawatase, Takaharu Hayashi, Satoki Nakamura, Nobuhiko Makino, Yoshiharu Higuchi

**Affiliations:** 1 Cardiovascular Medicine, Osaka Police Hospital, Osaka, JPN; 2 Postgraduate Clinical Training Center, Osaka Police Hospital, Osaka, JPN

**Keywords:** covid vaccination booster, booster, bnt162b2 mrna, covid-19 myopericarditis, covid-19 vaccine

## Abstract

Messenger ribonucleic acid (mRNA) vaccines against coronavirus disease 2019 (COVID-19) are highly effective in preventing and decreasing disease severity, but the duration of the effect is attenuated over time and repeated vaccination is required. A 41-year-old Japanese male presented to our hospital with chest pain three days after receiving the third dose of the BNT162b2 mRNA vaccine. After various examinations, such as endomyocardial biopsy and viral polymerase chain reaction (PCR) testing of endomyocardial biopsy tissue, we made the diagnosis of acute myopericarditis associated with booster vaccination. Here, we report a rare case of myopericarditis after booster mRNA vaccination.

## Introduction

Severe acute respiratory syndrome coronavirus 2 (SARS-CoV-2) was first reported in 2019 in China. It is the causative agent of coronavirus disease 2019 (COVID-19). The global COVID-19 pandemic has caused significant loss of life, profound disruption to lives and livelihoods, and widespread economic, sociological, and psychological damage. Active immunization with vaccination has been a critical mitigation tool against COVID-19. Emergency use authorization (EUA) for the first messenger ribonucleic acid (mRNA) vaccine, called BNT162b2 (Pfizer-BioNTech), was launched in December 2020 in the United Kingdom. Several clinical trials show that mRNA vaccines have high effectiveness in preventing infection and decreasing the severity of COVID-19 [1]. Vaccination against COVID-19 started in February 2021 in Japan. In light of reports of waning protection by six months after the primary two-dose vaccine series [2], the safety and efficacy of a third (booster) dose of the mRNA vaccine were evaluated [3]. As of December 2021, more than five million people have died of COVID-19. The third vaccination started in December 2021 in Japan.

Myocarditis is a well-known, rare complication of COVID-19 mRNA vaccination, especially in young adult and adolescent males. Recently, the Centers for Disease Control and Prevention (CDC) Advisory Committee on Immunization Practices identified a likely association between the COVID-19 mRNA vaccines from Pfizer-BioNTech and Moderna and cases of myocarditis and pericarditis [4].

A third dose of the COVID-19 vaccine has been proven to reduce confirmed infections and severe illnesses. There was no significant increase in the reporting of severe adverse events after the third dose of COVID-19 mRNA vaccines, such as anaphylaxis, cerebral venous sinus thrombosis, myocarditis, and pericarditis, compared with after earlier vaccine doses [3]. We report a rare case of myopericarditis in a patient who received the third dose of a COVID-19 mRNA vaccine two days before onset.

## Case presentation

A 41-year-old man diagnosed with asthma but currently not requiring treatment presented to our emergency department complaining of chest pain. He had developed a fever a couple of hours after receiving a booster dose of the BNT162b2 mRNA COVID-19 vaccine (Pfizer-BioNTech). He received the first dose of the BNT162b2 vaccine eight months prior and the second dose of the BNT162b2 vaccine seven months prior. He woke up from retrosternal chest pain two days after the booster dose vaccination. On the following day, his chest pain worsened, and he presented to our hospital.

**Figure 1:**
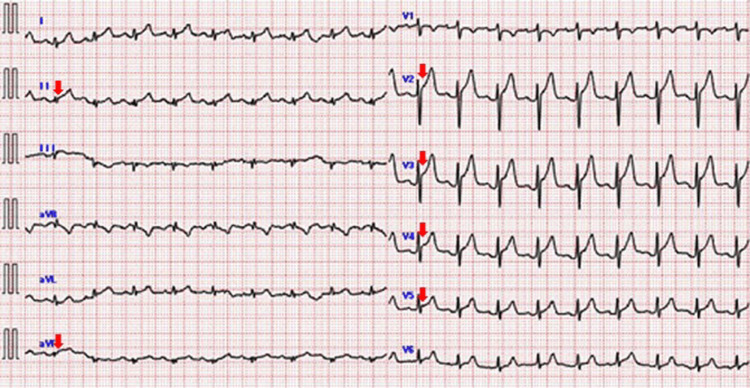
Electrocardiographic findings of ST-segment elevation, indicating inflammation of the epicardium Electrocardiography on admission showed significant ST-segment elevation in leads II, aVF, V2–5. The red arrows represent ST-segment elevation.

At presentation, the patient’s blood pressure was 138/100 mmHg, his pulse was 114 beats/min and regular, and oxygen saturation was 99% on room air. He had a fever of 37.4°C. On physical examination, no cardiac murmurs or friction rubs were noted on auscultation. His electrocardiogram revealed ST segment elevation in leads II, aVF, and V2-V5 (Figure 1).

Laboratory testing showed elevated levels of troponin T, creatine kinase, and inflammatory markers (Table 1).

**Table 1:** Laboratory findings The central column represents the reference range. Laboratory testing showed elevated levels of troponin T, creatine kinase, and inflammatory markers. WBC: white blood cells, RBC: red blood cells, Hb: hemoglobin, PLT: platelet, Neut: neutrophil, Eo: eosinophil, CK: creatine kinase, CK-MB: creatine kinase muscle and brain, AST: aspartate aminotransferase, ALT: alanine aminotransferase, LDH: lactate dehydrogenase, BUN: blood urea nitrogen, Cr: creatinine, CRP: C-reactive protein, SARS-CoV: severe acute respiratory syndrome coronavirus 2

Laboratory data (on admission)		
WBC (/μL)	3500-9800	14700
RBC (10*^6^/μL)	4.3-5.7	4.73
Hb (g/dL)	13.5-17.6	13.5
PLT (10*^3^/μL)	131-362	342
Neut (%)	30.0-75.0	86.1
Eo (%)	0-10	0.3
CK (U/L)	30-200	264
CK-MB (U/L)	25>	21
AST (U/L)	10-33	43
ALT (U/L)	6-35	27
LDH (U/L)	110-225	187
BUN (mg/dL)	8.4-20.4	12.8
Cr (mg/dL)	0.6-1.0	0.97
CRP (mg/dL)	8.8-10.4	6.23
Troponin T (ng/mL)	<0.1	0.318
SARS-CoV-2 PCR		negative
SARS-CoV-2 antigen		negative

**Figure 2:**
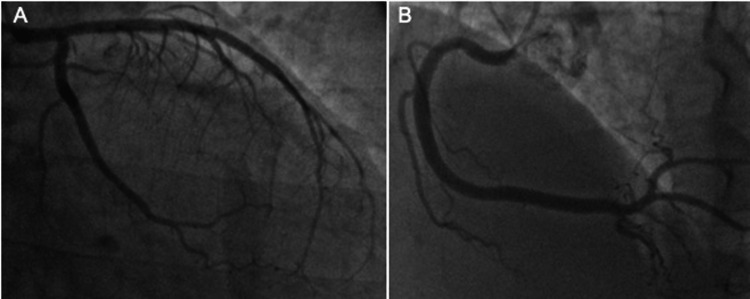
Coronary angiography revealed no significant stenosis Coronary angiography revealed no significant stenosis or occlusion in the (A) left coronary artery or the (B) right coronary artery.

Transthoracic echocardiography revealed hypokinesia in the posterolateral wall, a slightly reduced left ventricular ejection fraction of 50%, and no pericardial effusion. Emergent coronary angiography revealed no significant stenosis or occlusions in the coronary artery (Figures 2A-2B).

**Figure 3:**
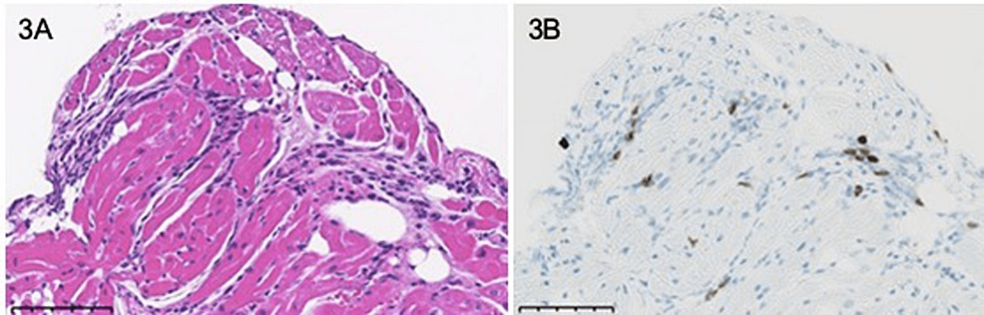
Pathological findings were compatible with myocarditis A: There was extensive inflammatory cell infiltration into the myocardium. The scale bar represents 100 μm. B: CD3 immunostaining. Inflammatory cells that invaded the myocardium included CD3-positive cells.

Rapid antigen and polymerase chain reaction (PCR) tests for SARS-CoV-2 were both negative (Table 1). We performed an endomyocardial biopsy and diagnosed myocarditis (Figures 3A-3B).

PCR tests from the myocardial tissue did not detect any viral genes (Table 2).

**Table 2:** Results of polymerase chain reaction tests from endomyocardial samples Polymerase chain reaction tests from the myocardial tissue did not detect any viral genes. DNA: deoxyribonucleic acid, RNA: ribonucleic acid

	viruses	result
DNA	Papillomaviruses	negative
	Parvoviruses	negative
	Herpes viruses	negative
	Hepatitis B virus	negative
RNA	Togaviruses	negative
	Enteroviruses	negative
	Flaviviruses	negative
	Orthomyxovirues	negative
	Paramyxoviruses	negative
	Coronaviruses	negative
	Rhabdoviruses	negative
	Hepatitis viruses	negative
	Lentivirus	negative

Based on all these clinical and laboratory data, myopericarditis associated with COVID-19 mRNA vaccination was diagnosed.

**Figure 4:**
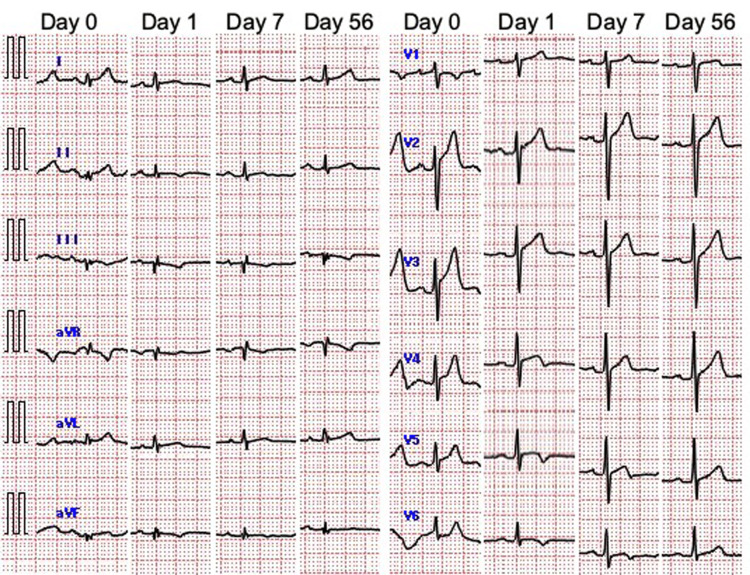
Changes in ECG from admission The ST-segment elevation at the time of admission became a negative T wave, relieved in one week, and almost normalized two months later.

The patient’s chest pain and fever resolved completely on day 3 without any medications. He was discharged on day 4. The ST-segment elevation led to the formation of negative T-waves after admission and gradually normalized (Figure 4).

Two months after discharge, echocardiography revealed fully restored cardiac function.

## Discussion

The incidence of mRNA vaccine-induced myocarditis after the primary two-dose vaccine series is reported to be approximately 9.3 in 1,000,000 overall, but it is approximately 52.4 in 1,000,000 among men between the ages of 12 and 25 years [5]. In most patients, the duration between vaccination and onset is approximately two to three days. Most cases of myocarditis following COVID-19 vaccination occur in young males, particularly following the second dose. The presentation is usually mild [5]. Our patient did not have a cold or diarrhea before vaccination. His clinical course was compatible with vaccine-associated myopericarditis with respect to the duration from vaccination to the onset and various examinations after admission. In our case, asthma was pointed out in childhood, but at present, there is no medication and no attacks have been recognized for more than 30 years. From this treatment course for asthma, we consider that the relationship between asthma and myopericarditis is poor in our case. It was consistent with previous reports in that most cases of vaccine-associated myocarditis occur in young, previously healthy patients. Since most cases of myocarditis following the mRNA vaccine have a mild clinical course, there were several reports that non-steroidal anti-inflammatory drugs, intravenous immunoglobulin, glucocorticoids, or colchicine were effective [6]. Rarely, they become fulminant and require immunosuppressants [7]. In our case, it is thought that relatively rapid improvement was achieved because lymphocyte infiltration was slight.

Historically, post-vaccination myocarditis has been reported as a rare adverse event and myopericarditis was most commonly reported after smallpox vaccination [8]. From our endomyocardial biopsy findings, it turned out to be lymphocytic myocarditis mainly composed of T lymphocyte infiltration. These findings were consistent with a previous report of vaccine-associated myocarditis [9]. Interferon-gamma production by type 1 helper T cells (Th1) following mRNA vaccination may play a role in cardiac inflammation [10]. Several pathogenic mechanisms have been proposed for vaccine-associated myocarditis, but none have been demonstrated. In the case of mRNA vaccines, nucleoside modifications reduce the mRNA's innate immunogenicity, but mRNA might still trigger an aberrant innate immune response. The excess reaction of the innate immune system against vaccine mRNA, initiating pro-inflammatory cascades through interleukin-1 and immunological pathways in the heart, might contribute to the pathophysiology of vaccine-associated myocarditis [11]. It has been reported that innate immunity also has immunological memory, and there might be an early reaction mediated by trained immunity that leads to myocarditis after the second or third vaccination [12]. Post-vaccination myocarditis might also be caused by acquired immunity induced by the active component of the spike protein of SARS-CoV-2 produced by mRNA-affected cells. Induced T cells and B cells might react against autoantigens that have some homology with the spike protein [11]. This immunological memory might cause an aberrant immune response upon receiving the second or third vaccine dose. Since the immune mechanism for the onset of myocarditis is unrevealed, it is difficult to assess whether myocarditis will develop again with the fourth vaccination or not. We do not feel like strongly recommending the fourth vaccination in our case.

Moreira et al. reported no cases of myocarditis or pericarditis among 5,081 participants who received a third BNT162b2 dose [3]. The rarity of myocarditis after booster vaccination might be attributed to the fact that patients who develop myocarditis after the first or second dose of mRNA vaccines are prohibited from receiving a booster dose of these mRNA vaccines. Sharff et al. estimated 9.1 cases of post-booster myopericarditis per 100,000 booster doses given in the relatively young age group of 18 to 39 years. In men, they estimated 14.7 cases per 100,000 booster doses given [13]. This risk is higher than previously estimated for two mRNA vaccine doses. It is unclear whether the frequency of myocarditis after booster vaccination is underestimated or overestimated; future reports are awaited. This is the first case report of a middle-aged Japanese man who developed acute myopericarditis after the third dose of the BNT162b2 vaccine.

## Conclusions

It is well-reported that myopericarditis following the COVID-19 vaccination is most likely to occur in male adolescents after the second dose of vaccination, but the frequency of myopericarditis after the third vaccination is unclear because it varies depending on the report. It is necessary to examine the effects of repeat mRNA vaccinations, and each adverse report is important because the COVID-19 pandemic is not fully contained. This is the first report in East Asia that reports a case who developed myopericarditis after the third dose of vaccination.

## References

[1] Polack FP, Thomas SJ, Kitchin N (2020). Safety and efficacy of the BNT162b2 mRNA COVID-19 vaccine. N Engl J Med.

[2] Thomas SJ, Moreira ED Jr, Kitchin N (2021). Safety and efficacy of the BNT162b2 mRNA Covid-19 vaccine through 6 months. N Engl J Med.

[3] Moreira ED Jr, Kitchin N, Xu X (2022). Safety and efficacy of a third dose of BNT162b2 COVID-19 vaccine. N Engl J Med.

[4] Bozkurt B, Kamat I, Hotez PJ (2021). Myocarditis with COVID-19 mRNA vaccines. Circulation.

[5] Oster ME, Shay DK, Su JR (2022). Myocarditis cases reported after mRNA-based COVID-19 vaccination in the US from December 2020 to August 2021. JAMA.

[6] Truong DT, Dionne A, Muniz JC (2022). Clinically suspected myocarditis temporally related to COVID-19 vaccination in adolescents and young adults: suspected myocarditis after COVID-19 vaccination. Circulation.

[7] Agdamag AC, Gonzalez D, Carlson K (2022). Fulminant myocarditis following coronavirus disease 2019 vaccination: a case report. Eur Heart J Case Rep.

[8] Mengesha B, Asenov AG, Hirsh-Raccah B, Amir O, Pappo O, Asleh R (2022). Severe acute myocarditis after the third (booster) dose of mRNA COVID-19 vaccination. Vaccines (Basel).

[9] Chow BT, Lai CK (2022). Lymphohistiocytic myocarditis possibly due to Moderna mRNA-1273 vaccine. Am J Clin Pathol.

[10] Hajjo R, Sabbah DA, Bardaweel SK, Tropsha A (2021). Shedding the light on post-vaccine myocarditis and pericarditis in COVID-19 and non-COVID-19 vaccine recipients. Vaccines (Basel).

[11] Teijaro JR, Farber DL (2021). COVID-19 vaccines: modes of immune activation and future challenges. Nat Rev Immunol.

[12] Yao Y, Jeyanathan M, Haddadi S (2018). Induction of autonomous memory alveolar macrophages requires T cell help and is critical to trained immunity. Cell.

[13] Sharff KA, Dancoes DM, Longueil JL, Lewis PF, Johnson ES (2022). Myopericarditis after COVID-19 booster dose vaccination. Am J Cardiol.

